# High pressure study of sodium trihydride

**DOI:** 10.3389/fchem.2023.1306495

**Published:** 2024-01-09

**Authors:** Tomas Marqueño, Mikhail A. Kuzovnikov, Israel Osmond, Phillip Dalladay-Simpson, Andreas Hermann, Ross T. Howie, Miriam Peña-Alvarez

**Affiliations:** ^1^ Centre for Science at Extreme Conditions (CSEC), The School of Physics and Astronomy, The University of Edinburgh, Edinburgh, United Kingdom; ^2^ Center for High Pressure Science and Technology Advanced Research, Shanghai, China

**Keywords:** hydrides, sodium, high pressure, X-ray diffraction, Raman, density functional calculations

## Abstract

The reactivity between NaH and H_2_ has been investigated through a series of high-temperature experiments up to pressures of 78 GPa in diamond anvil cells combined with *first principles* calculations. Powder X-ray diffraction measurements show that heating NaH in an excess of H_2_ to temperatures around 2000 K above 27 GPa yields sodium trihydride (NaH_3_), which adopts an orthorhombic structure (space group *Cmcm*). Raman spectroscopy measurements indicate that NaH_3_ hosts quasi-molecular hydrogen ( ${\text{H}}_{2}^{\delta -}$ ) within a NaH lattice, with the ${\text{H}}_{2}^{\delta -}$ stretching mode downshifted compared to pure H_2_ (Δ*ν* ∼−120 cm^−1^ at 50 GPa). NaH_3_ is stable under room temperature compression to at least 78 GPa, and exhibits remarkable *P-T* stability, decomposing at pressures below 18 GPa. Contrary to previous experimental and theoretical studies, heating NaH (or NaH_3_) in excess H_2_ between 27 and 75 GPa does not promote further hydrogenation to form sodium polyhydrides other than NaH_3_.

## 1 Introduction

The synthesis of metallic alloys hosting hydrogen at high pressures has attracted significant interest, especially after the suggestion of their potential high-temperature superconductivity (Ashcroft, 2004). This was demonstrated by the pioneering work on LaH_10_ with a critical temperature (*T*_*c*_) of *T*_*c*_ = 250 K at 170 GPa (Drozdov et al., 2019; Somayazulu et al., 2019). However, there are essential aspects of high pressure hydrides that must be understood in order to provide an efficient route towards desired target properties. The alkali metal lithium was one of the first systems studied as a promising hydrogen host for superconductivity (Zurek et al., 2009). However, experimentally these compounds have proven difficult to synthesize, with some spectroscopic evidence that LiH reacts with H_2_ and transforms into LiH_2_ and LiH_6_ above 130 GPa hosting molecular hydrogen, which would prevent any favorable electron phonon coupling (Howie et al., 2012; Pépin et al., 2015).

Subsequent *first principles* simulations predicted that all of the heavier alkali metals could form highly stoichiometric hydrides (Baettig and Zurek, 2011; Hooper and Zurek, 2012; Shamp et al., 2012; Hooper et al., 2013; Shipley et al., 2021). Some of them such as KH_*n*_ with *n* > 1 could also form stable polyhydrides at pressures as low as 3 GPa (Hooper and Zurek, 2012). Despite these theoretical predictions, only the hydrides of lithium (Pépin et al., 2015) and sodium (Struzhkin et al., 2016) have been experimentally approached. This is due to the experimental difficulties arising from studying alkali elements, which instantly react with the oxygen and water present in the atmosphere. The high reactivity of the alkali metals is the cause why there are very few studies on their polyhydrides.

Structural searches suggest that alkali metal polyhydrides are some of the best candidates for high *T*_*c*_ superconductivity among all possible binary hydrides (Shipley et al., 2021), with high predicted T_*c*_ values at lower pressures than those required for previously observed superconducting rare earth hydrides. In particular, superconductivity is predicted in Na_2_H_11_ (*T*_*c*_ ∼ 150 K at 100 GPa) and in NaH_6_, where *T*_*c*_ predictions range from 180 to 260 K at 50 and 100 GPa respectively (Chen et al., 2021; Shipley et al., 2021). The Na-H system has previously been studied experimentally up to 50 GPa, forming NaH_3_ (*Cmcm*) and NaH_7_ (*Cc*) above 40 GPa and 2000 K Struzhkin et al. (2016). Analogous to the lithium polyhydrides, NaH_3_ and NaH_7_ have been described to host quasi-molecular hydrogen. Thus, the sodium-hydrogen system remains experimentally unexplored above 50 GPa.

In this work, we explore the reactivity of the NaH-H_2_ system up to pressures of ∼78 GPa and temperatures in excess of 2000 K using X-ray diffraction (XRD) and Raman spectroscopy diagnostics, combined with *first principles* calculations. Laser heating NaH in an excess H_2_ medium at pressures above 27 GPa yields *Cmcm*-NaH_3_, which remains the only product up to pressures of 78 GPa. The Raman spectrum of NaH_3_ is dominated by an intense mode (at ∼4120 cm^−1^) related to the intramolecular stretching modes of quasi-molecular ${\text{H}}_{2}^{\delta -}$ units within the NaH_3_ structure. Relative to pure H_2_ (Sharma et al., 1980; Howie et al., 2013), this mode is at lower frequencies, and upshifts monotonically in frequency up to 75 GPa, indicating a shortening of the H-H bonds, with no evidence of change in its pressure-frequency dependence. NaH_3_ exhibits remarkable pressure-temperature stability, decomposing into hydrogen and sodium monohydride upon room temperature decompression below 18 GPa.

## 2 Experimental details

Diamond anvil cells were prepared with diamond culet sizes of 80–200 *μ*m, with the sample chamber formed by pre-indentation and laser drilling of a Re gasket. Sodium monohydride (Sigma Aldrich, 95% dry) was loaded into the sample chambers in an inert argon atmosphere glove box to prevent the oxidation of NaH. This was loaded together with small grains of gold powder used as a pressure calibrant for XRD experiments (Fei et al., 2007). The DACs were subsequently loaded with research grade (99.9999%) hydrogen gas at 2000 bar. The samples were heated using a 1,064 nm YAG laser. A summary of the experiments performed with each sample is given in Supplementary Table S1 in the Supplementary Data.

The XRD measurements were performed at the Sector 13 (GSECARS) beamline at the Advanced Photon Source (ANL, United States) (Shen et al., 2005; Prakapenka et al., 2008) and the P02.2 Extreme Conditions Beamline at PETRA-III (DESY, Germany) (Liermann et al., 2015). The relevant details of the beamline regarding our experiments are summarized in Supplementary Table S2 (Supplementary Data). The integration of the XRD patterns was performed using DIOPTAS (Prescher and Prakapenka, 2015). The analysis and refinement of the obtained XRD patterns were carried out using the software packages PowderCell and GSAS-II (Kraus and Nolze, 1996; Toby and Von Dreele, 2013).

Raman measurements of the samples were acquired using a custom-built confocal microfocused Raman setup with 514.5 nm Ar-ion laser excitation source, equipped with a PyLoN:100 cryogenically cooled CCD camera (1,340 × 100 sensor). In order to measure Raman spectra in a wide range of frequencies, a grating of 300 lines/mm was used. Pressure was monitored in Raman measurements using the frequency of the vibron of excess H_2_ (Howie et al., 2013), and cross-checked with the frequency of the stressed Raman edge of diamond (Akahama and Kawamura, 2006).

## 3 Computational details

Total energy density functional theory calculations were performed with the VASP package, using plane wave basis sets in conjunction with projector augmented wave (PAW) atomic data sets (Kresse and Furthmüller, 1996; Kresse and Joubert, 1999). The plane wave energy cutoff was 800 eV, Brillouin zone sampling used regular grids with linear k-point densities of 40/Å^−1^, and ‘hard’ PAW datasets that consider the 1*s* ^1^/2*s* ^2^2*p* ^6^3*s* ^1^ states as valence electrons, and have cutoff radii 0.8/2.2 Bohrs for H/Na, respectively. The structures were fully optimized over a range of pressures between 10 and 90 GPa, and until all atomic force components were below 1 meV/Å. Electronic exchange-correlation effects were described within the generalized gradient approximation, using the Perdew–Burke–Ernzerhof (PBE) parameterization (Perdew et al., 1996) and, for comparison, the semi-empirical Grimme D3 dispersion correction (Grimme et al., 2011) as well as the nonlocal vdW-DF optB88-vdW functional (Klimeš et al., 2010) (Supplementary Data, Supplementary Figure S1). Phonon calculations were performed using the finite-displacement method in suitable supercells of all Na-H compounds considered here (see Supplementary Material for details, Supplementary Figure S2); they were set up and analyzed using the phonopy and phonopy-spectroscopy packages (Togo and Tanaka, 2015; Skelton et al., 2017). Reaction enthalpies between NaH, NaH_6_, H_2_ to form NaH_3_ are shown in Supplementary Figure S3 (Supplementary Data).

## 4 Results

### 4.1 X-ray diffraction experiments

**FIGURE 1:**
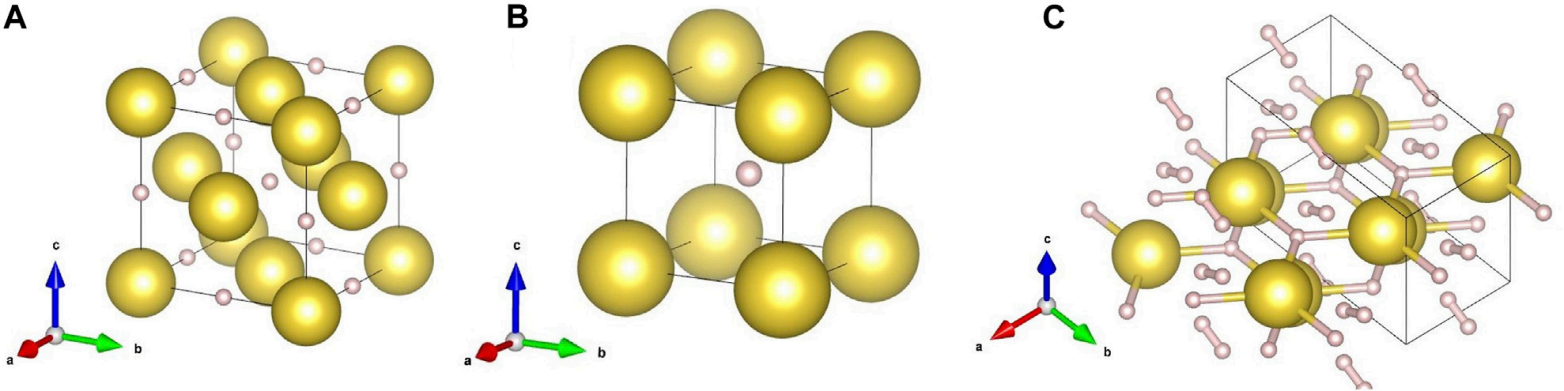
Relevant crystal structures of the Na-H system in our study: **(A)** NaH-I $\left(Fm\bar{3}m\right)$ , **(B)** NaH-II $\left(Pm\bar{3}m\right)$ and **(C)** NaH_3_ (*Cmcm*). Yellow and white spheres represent Na and H atoms, respectively. The structures were taken from Shull et al. (1948), Duclos et al. (1987) and Struzhkin et al. (2016).

At room pressure and temperature, sodium hydride (NaH-I) crystallizes in a *fcc* structure analogous to that of NaCl (rock salt), in which hydrogen atoms occupy octahedral interstitial sites (Shull et al., 1948). Above ∼30 GPa, NaH undergoes a phase transition from a *fcc* to a CsCl-type (simple cubic, NaH-II) structure (Duclos et al., 1987). Both NaH-I and II contain atomic hydrogen, which partially forms ionic bonds with sodium. The unit-cells of these NaH-I and II structures are shown in Figures 1A, B respectively.

**FIGURE 2:**
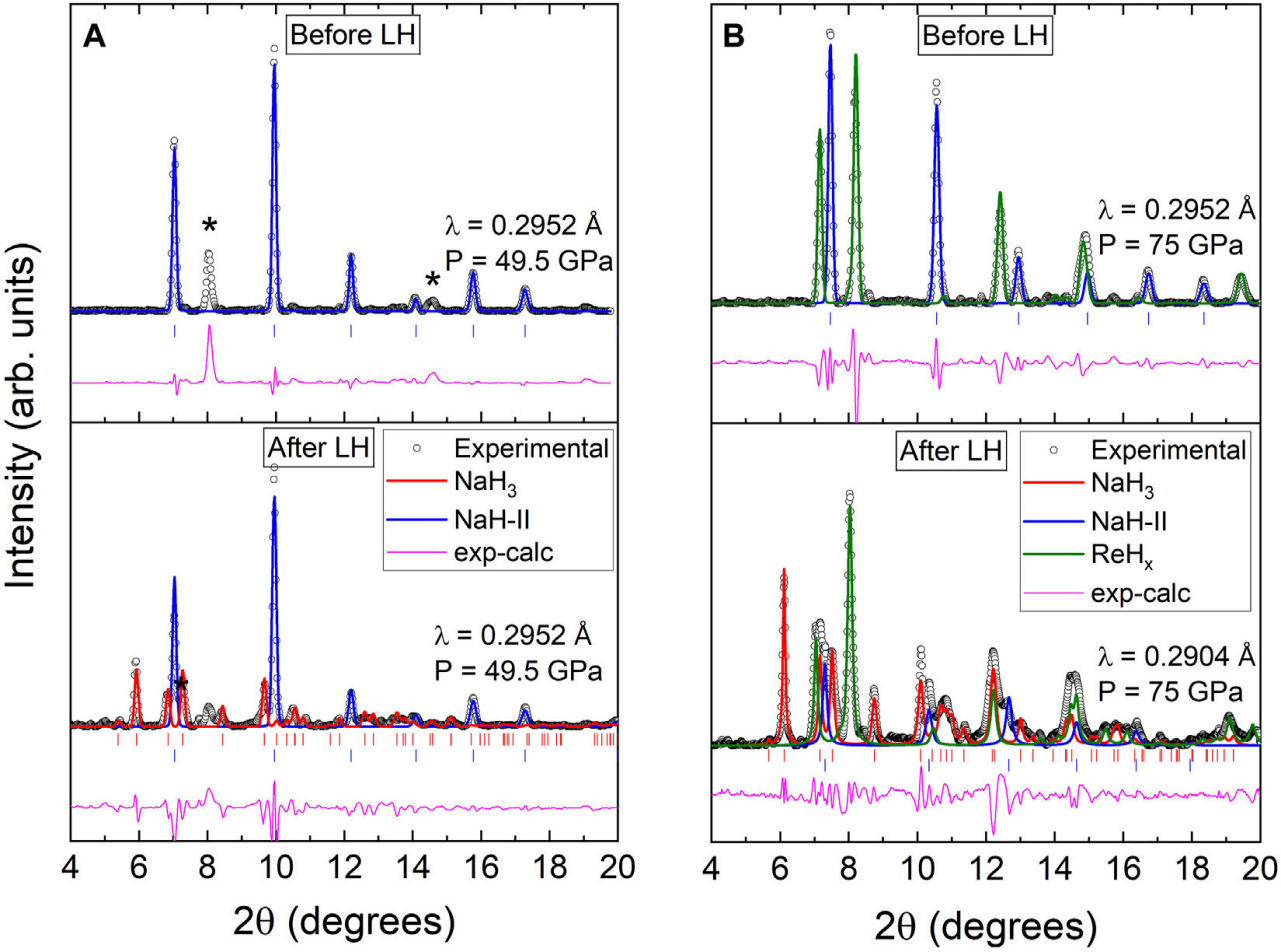
**(A)** *&* **(B)** Powder XRD pattern and LeBail refinements of two different samples before and after laser heating at 49.5 GPa and 75 GPa, respectively. Experimental data is represented by circles. The calculated contributions from NaH-II, *Cmcm*-NaH_3_ and hcp-ReH_*x*_ phases are shown by the blue, red, and green curves, respectively. The reflections corresponding to NaH_3_ and NaH are indicated with red and blue ticks respectively. Asterisks in **(A)** indicate reflections associated with ReH_*x*_.

**FIGURE 3:**
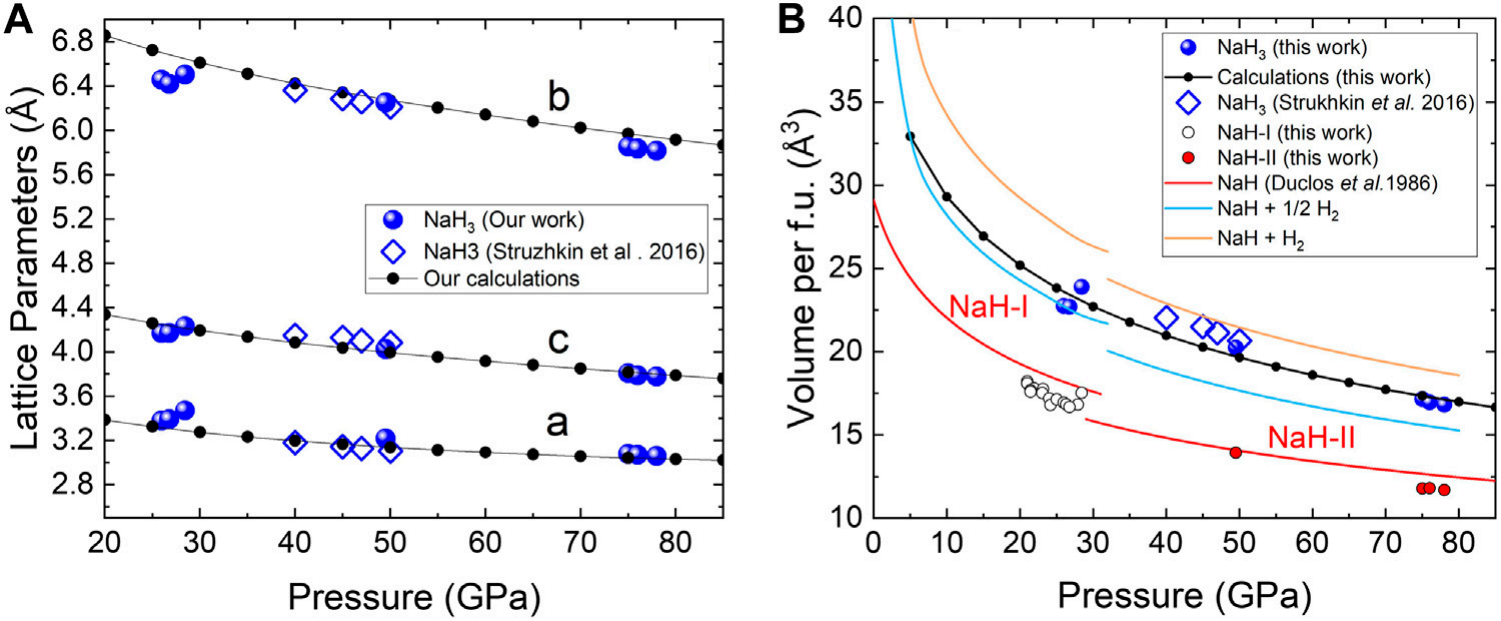
Pressure dependence of the **(A)** lattice parameters and **(B)** volume per formula unit of NaH_3_. The data obtained in our experiments is represented with blue spheres; small connected black dots stand for the results of our calculations. The data reported by Struzhkin et al. (2016) is depicted with diamonds. The red solid line stands for the volume-pressure curves of NaH (phases I and II) reported by Duclos et al. (1987). Blue and orange solid lines stand for the volume sums *V*(NaH) + $\frac{1}{2}V$ (H_2_) and *V*(NaH) + *V*(H_2_), respectively (Duclos et al., 1987; Loubeyre et al., 1996).

NaH in H_2_ was compressed to 50 GPa and we did not observe the formation of any higher hydride with a larger hydrogen content, as shown in the bottom panel of 2 a). Since there are likely to be energy barriers preventing the chemical reaction between NaH and H_2_, we laser heated our sample at ∼50 GPa until thermal emission was visible. The estimated maximum temperatures on the laser-heated surface range from 1,500 to 2100 K, according to the thermal emission of blackbody radiation from the sample. As shown in Figure 2A, the quenched sample is mainly composed of non-reacted NaH-I (*fcc*) and a second phase which we identify as *Cmcm*-NaH_3_. The unit-cell of NaH_3_ is shown in Figure 1C. NaH_3_ has a distorted hexagonal close-packed (*hcp*) lattice of sodium atoms. According to the DFT-based *first principles* predictions (Struzhkin et al., 2016), the hydrogen in NaH_3_ is present in the form of H^−^ anions with planar triangular coordination within the *hcp* planes of metal atoms, and ${\text{H}}_{2}^{\delta -}$ quasi-molecular units filling the octahedral interstitial sites. At 49.5 GPa, the lattice parameters of NaH_3_ after the LeBail refinement are *a* = 3.218 Å, *b* = 6.251 Å and *c* = 4.027 Å (*V*_f.u._ = 20.25 Å^3^). The evolution of the lattice parameters and unit-cell volume are in reasonably good agreement with the observations reported by Struzhkin et al. (2016) and our own DFT calculations (Figure 3). As further verification for the NaH_3_ stoichiometry, we estimate the minimum amount of hydrogen using the Le Châtelier principle regarding volume change:

$$V\left({\text{NaH}}_{n}\right)\leq V\left(\text{NaH}\right)+\left(n-1\right)V\left(\frac{1}{2}{\text{H}}_{2}\right)$$ (1)

in which we can use the volumes of NaH and H_2_ reported in the literature (Duclos et al., 1987; Loubeyre et al., 1996). The sum of the individual volumes per formula unit (f.u.) of NaH and H_2_ is displayed in Figure 3B for *n* = 1, 2 and 3 (Eq. 1). Experimental volumes are above and below the curves corresponding to *n* = 2 and 3, respectively. Thus, both XRD patterns and experimental volumes support the assignment to *Cmcm*-NaH_3_.

To investigate the stability range of NaH_3_, we heated subsequent samples below 50 GPa to temperatures in excess of 2000 K, where synthesis could be achieved as low as 30 GPa (see Supplementary Figure S4 in the Supplementary Data). Heating NaH + H_2_ at 75 GPa to similar temperatures converted a greater proportion of NaH to NaH_3_ than heating at either 27 GPa or 49.5 GPa. This could indicate greater stability and lower kinetic barriers to formation at higher pressure, but could also be due to differences in sample morphology. However, we did not observe any signatures of any other polyhydride compounds upon laser heating in the 30–75 GPa pressure range, with traces of the already reported NaH_7_ polyhydride (Struzhkin et al., 2016). These results agree with our theoretical predictions, which indicate that within the experimentally explored conditions NaH_3_ should form from NaH and H_2_, irrespective of exchange-correlation functional used (Supplementary Data, Supplementary Figures S1–S3). In the case of recently suggested NaH_6_ (Chen et al., 2021; Shipley et al., 2021), our calculations suggest that this polyhydride should react with NaH to form NaH_3_ at any pressure below 100 GPa (Supplementary Figure S3).

### 4.2 Raman scattering experiments

**FIGURE 4:**
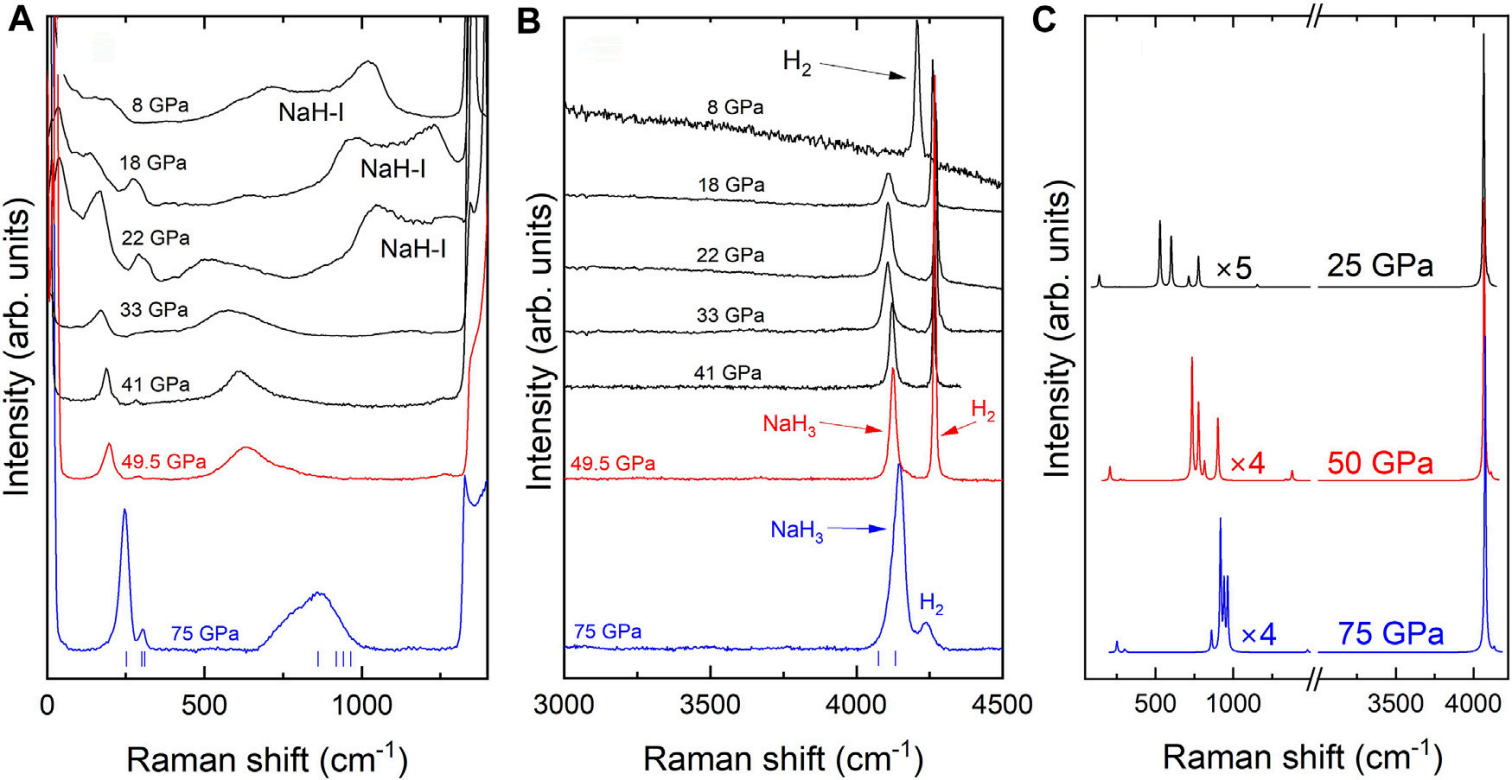
**(A,B)** Raman spectra of NaH_3_ samples. Blue and red solid lines correspond to NaH_3_ samples at 75 and 49.5 GPa, respectively, which were discussed in Figure 2. The black solid lines correspond to a different Raman experiment on NaH_3_ upon decompression from 41 to 8 GPa. Blue vertical ticks represent the simulated Raman frequencies of NaH_3_ at 75 GPa. **(C)** Simulated Raman spectra of NaH_3_ at 75, 50 and 25 GPa (blue, red and black lines, respectively). The peak intensities of the spectra below 1,500 cm^−1^ have been magnified by the indicated factors.

Prior to the heating of the sample, NaH-II exhibited no Raman activity, as it is shown in Supplementary Figure S6 (Supplementary Data) for a sample at ∼50 GPa. After laser heating, the Raman spectrum changes significantly, with intense activity attributed to the formation of NaH_3_. Figures 4A, B show the spectra of different samples of NaH_3_ at different pressures. The spectral distribution of the modes is in good agreement with our simulated Raman spectra for NaH_3_, which are shown in Figure 4C. Remarkably, the Raman signatures of NaH_3_ are experimentally observed down to 18 GPa upon decompression, with the absence of any Raman activity below this pressure suggesting that the decomposition products are NaH and H_2_.

At frequencies below 1,200 cm^−1^, the Raman spectrum of NaH_3_ at 75 GPa is characterized by an intense, sharp mode at ∼ 250 cm^−1^, together with a lower intensity satellite mode at ∼ 300 cm^−1^ and a broad band at ∼800 cm^−1^. Our DFT calculations relate the first two bands to a B_g_ mode, and to the overlap of an A_g_ and B_3g_ modes respectively. Both bands are then associated with Na and atomic H displacements in opposite directions coupled to one H_2_ libration. The broad band at ∼ 800 cm^−1^ can be ascribed to the overlap of four Raman modes: B_3g_, B_1g_, B_2g_ and A_g_. These modes involve both librational and translational vibrations of the quasi-molecular ${\text{H}}_{2}^{\delta -}$ units, coupled with displacements of the atomic hydrogen, while the Na atoms remain almost stationary. On decompression, below 30 GPa, we observe the growth of wide contributions between 1,000 and 1,300 cm^−1^, Figure 4A. Our XRD results do not show any symmetry change due to possible structural transitions in NaH_3_, which remains *Cmcm* down to 18 GPa, Therefore, since these new bands appear after the transition from NaH-II (*sc*) to NaH-I (*fcc*), they could be related to second-order Raman modes of NaH-I, similar to those observed in isomorphic LiH (*fcc*), so we labelled them as “NaH-I”. (Ho et al., 1997; Howie et al., 2012).

**FIGURE 5:**
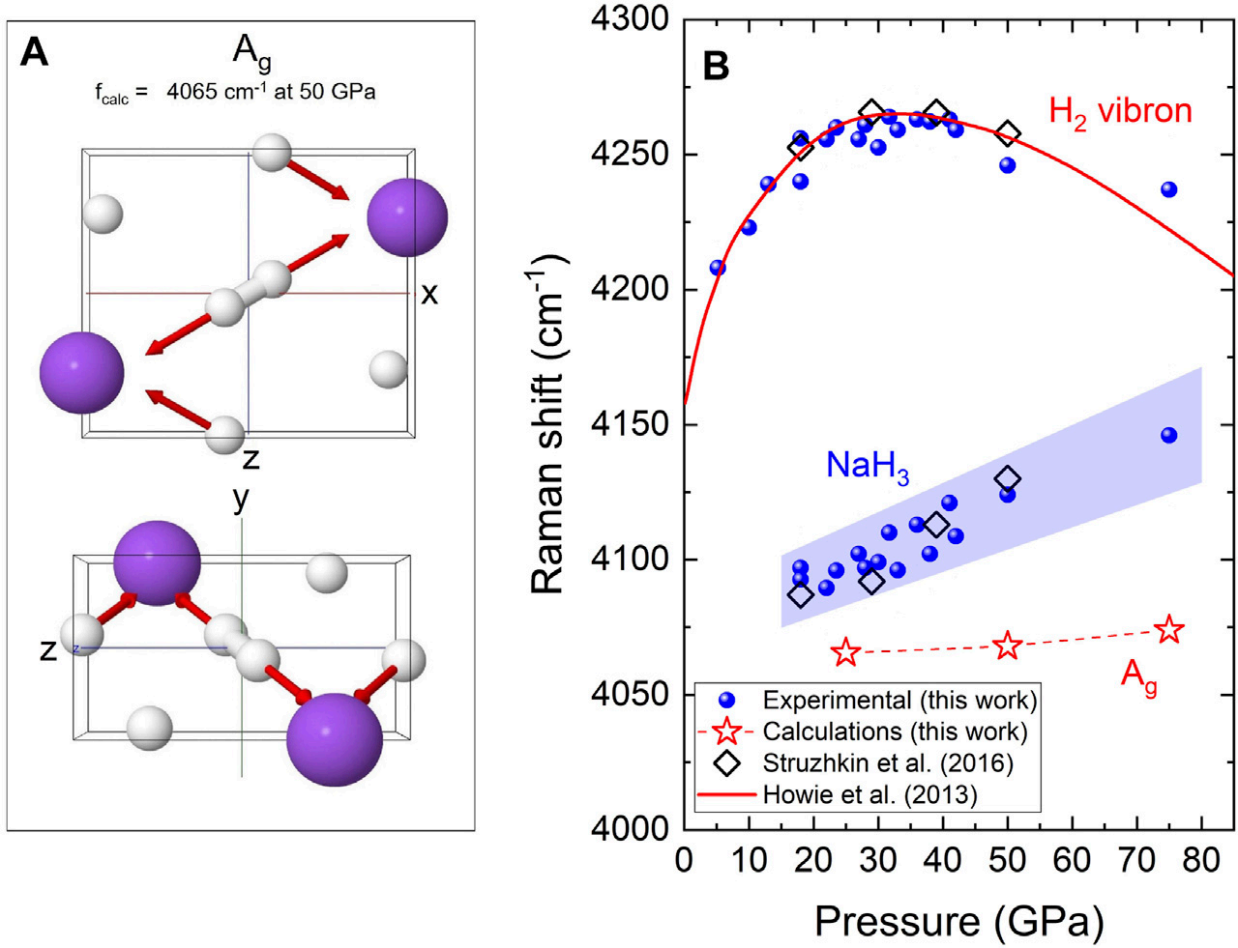
**(A)** Eigenmode corresponding to the ${\text{H}}_{2}^{\delta -}$ vibron of NaH_3_ under different perspectives according to primitive unit-cell coordinates. Na and H atoms are represented by purple and white spheres, respectively. The red arrow represents the motion associated with each atom. The calculated frequency *f*_calc_ is 4,065 cm^−1^ at 50 GPa. **(B)** Pressure evolution of the vibron frequencies of H_2_ and NaH_3_. Our experimental data is represented with blue spheres, with the area shaded in blue depicting the FWHM of the peaks associated with NaH_3_. Calculated data is shown with connected stars. Dashed lines are for visual guidance. Diamonds represent the experimental data reported by Struzhkin et al. (2016). The pressure evolution of the pure H_2_ vibron reported by Howie et al. (2013) is shown as a solid red line.

At high frequency, the Raman spectrum of NaH_3_ is dominated by an intense mode at 4,145 cm^−1^ (at 75 GPa), which is directly related to the H-H stretching vibrations from quasi-molecular ${\text{H}}_{2}^{\delta -}$ units in NaH_3_. In hydride systems containing H_2_ units, the surrounding metallic lattice and the resulting charge transfer leads to modified H-H bond lengths when compared to that of pure hydrogen, causing a frequency downshift in the ${\text{H}}_{2}^{\delta -}$ vibron (Peña-Alvarez et al., 2022). In this frequency regime, two Raman modes are predicted to be present at similar frequencies, A_g_ and B_3g_. However the intensity of the B_3g_ is expected to be significantly lower, i.e., *I*(B_3g_)/*I*(A_g_) ∼ 1.2%, according to our calculations. The eigenmode of the A_g_ vibration is depicted in Figure 5A showing its correspondence to the H-H stretching mode.

Interestingly, some NaH samples exhibited an intense photoluminescence background signal after exposure to X-ray radiation and IR laser used for heating. This can be attributed to the formation of color centers, which have previously been observed in studies of pure NaH Duclos et al. (1987); Williams (1962) and reported for isoelectronic alkaline halides such as NaF (Bryukvina et al., 2014). Furthermore, whilst the samples undergo a visual color change, appearing orange after X-ray irradiation at 75 GPa (Supplementary Figure S5B [Supplementary Data]), XRD mapping of the samples showing this photoluminescence did not reveal the formation of any other product or phase transition.

Figure 5B presents the Raman shift as a function of pressure of the NaH_3_ ${\text{H}}_{2}^{\delta -}$ vibron, from our experimental and theoretical results, together with that of pure H_2_ (Howie et al., 2013). Comparing the Raman spectra of NaH_3_ and H_2_, we observe that the quasi-molecular ${\text{H}}_{2}^{\delta -}$ vibron is at lower frequencies than that of pure hydrogen. For instance, at 50 GPa in NaH_3_ the ${\text{H}}_{2}^{\delta -}$ vibron is at 4,124 cm^−1^, whereas in pure H_2_ the vibron is at 4,246 cm^−1^ (Δ*ν* = −122 cm^−1^). It is known that the compression of hydrogen leads to an initial upshift of its vibron, up to a maximum at 30–40 GPa, after which intermolecular interactions lead to a vibron downshift upon further compression (Sharma et al., 1980; Howie et al., 2012; Howie et al., 2013). In NaH_3_, however, the ${\text{H}}_{2}^{\delta -}$ vibron monotonically upshifts upon compression, going from 4,092 cm^−1^ at 18 GPa to 4,146 cm^−1^ at 75 GPa. This indicates that, in contrast to pure H_2_, pressure does not favor the lengthening of the H-H bond in NaH_3_, which is responsible for the eventual vibron downshift in pure hydrogen.

Similar behavior is also observed in previous measurements on the lithium-hydride system. IR absorption measurements show that while the vibron of pure H_2_ is located at around 4,450 cm^−1^ at 240 GPa (Zha et al., 2013), the vibrons of LiH_2_ and LiH_6_ are around 3900 cm^−1^ and 2,600 cm^−1^ at 160 GPa, respectively (Pépin et al., 2015). Like NaH_3_, these vibrons in LiH_2_ and LiH_6_ display a positive frequency shift with pressure. For instance, in LiH_2_ the vibron upshifts from 3900 at 160 GPa to 4,250 cm^−1^ at 220 GPa. These results contrast with the experimental observations on alkaline earth tetrahydrides (MH_4_ with M = Ca, Sr, Ba) which also contain quasi-molecular hydrogen (Peña-Alvarez et al., 2022). In these tetrahydrides, the frequency of the ${\text{H}}_{2}^{\delta -}$ vibron downshifts upon compression, which is directly related to a progressive elongation of the intramolecular bond.

The pressure induced H-H lengthening in binary hydrides has been suggested to be directly related to the charge transfer from the host element to the ${\text{H}}_{2}^{\delta -}$ unit, populating the σ* orbitals (Wang et al., 2012; Bi and Zurek, 2021). This would lead to an increase in the repulsion of the anti-bonding orbitals and the subsequent weakening of the bond. However, more recent studies have shown that the stretching of the H-H bond under compression results from a combination of charge transfer and the confinement of the molecule to small interstitial locations (Peña-Alvarez et al., 2022).

According to our calculations, shown in Table 1, the charge of the Na cation goes from +0.7842 at 25 GPa to +0.7490 at 75 GPa. Similarly, the charge of the H anions goes from −0.6690 to −0.6274 at 25 and 75 GPa, respectively. Thus, most of the charge is transferred from sodium to the monoatomic hydrogen, while there is some charge on the ${\text{H}}_{2}^{\delta -}$ quasi-molecular units, which remains almost constant over this pressure range (*δ* ∼ 0.12). With very little charge transfer into the ${\text{H}}_{2}^{\delta -}$ units, there is no substantial increase in the occupation of the antibonding states of the ${\text{H}}_{2}^{\delta -}$ units, which is compatible with the shortening of the H-H quasi-molecular bond upon compression. Factors such as the relative arrangement of the elements within the crystal structure and the size of the cation are also expected to play an important role in the observed pressure dependence (Peña-Alvarez et al., 2022). For example, in NaH_3_, the ${\text{H}}_{2}^{\delta -}$ units are located between hexagonal layers of Na-H, whereas in alkali-earth tetrahydrides ${\text{H}}_{2}^{\delta -}$ units occupy the interstitial positions of 3D-compact network structures (Peña-Alvarez et al., 2022). Therefore, our observations on the quasi-molecular Raman vibron of NaH_3_ can be justified by 1) the constant charge of the ${\text{H}}_{2}^{\delta -}$ units and 2) the location of the ${\text{H}}_{2}^{\delta -}$ units between Na-H layers.

**TABLE 1:** Calculated average charges for sodium, quasi-molecular and atomic hydrogen inside of the *Cmcm*-NaH_3_ structure at different pressures.

Pressure (GPa)	Na	$H_{2}^{\delta -}$ units	Atomic H
25	+0.7842	−0.1153	−0.6690
50	+0.7669	−0.1173	−0.6496
75	+0.7490	−0.1216	−0.6274

The results presented here show that the previous study on the Na-H misinterpreted their Raman spectra by attributing the characteristic A_g_ mode of *Cmcm*-NaH_3_ to *Cc*-NaH_7_ (Struzhkin et al., 2016). Furthermore, the Raman modes assigned to NaH_3_ by Struzhkin et al. (2016) can be explained by the formation of CH_4_-H_2_ compounds from the high temperature reaction between the diamond anvil and the H_2_ medium (Peña-Alvarez et al., 2021; Ranieri et al., 2022). Our XRD and Raman results provide no evidence for the existence of *Cc*-NaH_7_ in the 25–75 GPa pressure range, which is within the synthesis conditions previously reported. We can as well rule out the formation of any of the predicted sodium polyhydrides (Baettig and Zurek, 2011; Chen et al., 2021; Shipley et al., 2021), to at least 78 GPa.

## 5 Conclusion

We have synthesized NaH_3_ from NaH and H_2_ at the high hydrogen pressures of ∼ 30, 40, 50 and 75 GPa and temperatures of 2000 K using laser heating. XRD measurements at 27, 49.5 and 75 GPa show that the crystal structure of the metal sublattice of NaH_3_ is compatible with the orthorhombic space group *Cmcm*. The Na volume expansion corresponds to a NaH_3_ stoichiometry. The unit-cell lattice parameters and unit-cell volume experimental points as a function of pressure are in good agreement with our DFT calculations for NaH_3_ and the previously reported data below 50 GPa (Struzhkin et al., 2016). Different and independent samples of NaH_3_ at 75 and 50 GPa were measured by Raman spectroscopy. Further Raman experiments were performed on an additional sample to characterize NaH_3_ upon decompression from 41 to 8 GPa. The NaH_3_ Raman spectrum is characterized by a phonon near 4,125 cm^−1^ ( ∼50 GPa), assigned to the stretching vibration of quasi-molecular ${\text{H}}_{2}^{\delta -}$ units trapped inside of the structure, visible at lower frequencies than that of pure H_2_. In contrast to pure H_2_, the stretching mode from ${\text{H}}_{2}^{\delta -}$ units in NaH_3_ upshifts during compression, indicating that chemical pre-compression is not favored. Despite several experimental attempts, we were unable to identify any sodium polyhydride with a hydrogen content higher than NaH_3_, suggesting that NaH_3_ may be the only stable hydride below 78 GPa.

## Data Availability

The raw data supporting the conclusion of this article will be made available by the authors, without undue reservation.
