# Supplementary material for: High pressure study of sodium trihydride
Source: Front Chem. 2024 Jan 9;11:1306495. doi: 10.3389/fchem.2023.1306495 (PMC10803492; doi:10.3389/fchem.2023.1306495)
Supplement: Supplementary file 1 [file DataSheet1.pdf]

Supplementary Material

1 EXPERIMENTAL DETAILS

Table S1. Summary of the procedures followed for each NaH+H_2 sample.

**Diamond culet size** (μm)	**Procedure**
200	(1) Compress to $\sim$ 50 GPa under Raman.<br>(2) Laser Heat<br>(3) Measure Raman spectrum [Fig. 4 (a) and (b), red line]<br>(4) Measure XRD patterns at 50 GPa [Fig. 2 (a)].
80	(1) Compress to $\sim$ 75 GPa under XRD.<br>(2) Laser Heat<br>(3) Measure XRD patterns [Fig. 2 (b)]<br>(4) Measure Raman spectra at 75 GPa [Fig. 4 (a) and (b), blue line].<br>(5) Compress further to 80 GPa while measuring XRD patterns.
200	(1) Compress to $\sim$ 30 GPa under X-rays.<br>(2) Laser Heat<br>(3) Measure XRD patterns [Fig. S4]<br>(4) Decompress to 25 GPa while measuring XRD patterns.
200	(1) Compress to $\sim$ 40 GPa under Raman.<br>(2) Laser Heat<br>(3) Measure Raman spectra under decompression down to 8 GPa [Fig 4 (a) and (b), black lines].

Table S2. Details of the diffraction beamlines in which the experiments were performed.

**Synchrotron**	**Details**
Advanced Photon Source (APS)	Beamline: 13 IDD - GSECARS<br>$\lambda_1 = 0.2952$ Å (monochromatic beam)<br>$\lambda_2 = 0.4066$ Å (monochromatic beam)<br>Beam spot size: 5×5 μm^2<br>Detector (2D): Eiger 500K
Deutsches Elektronen-Synchrotron (DESY)	Beamline: P02.2 Extreme Conditions Beamline<br>$\lambda_1 = 0.2904$ Å (monochromatic beam)<br>Beam spot size: 2×2 μm^2<br>Detector (2D): Perkin Elmer (XRD1621)

2 SUPPLEMENTARY COMPUTATIONAL DATA

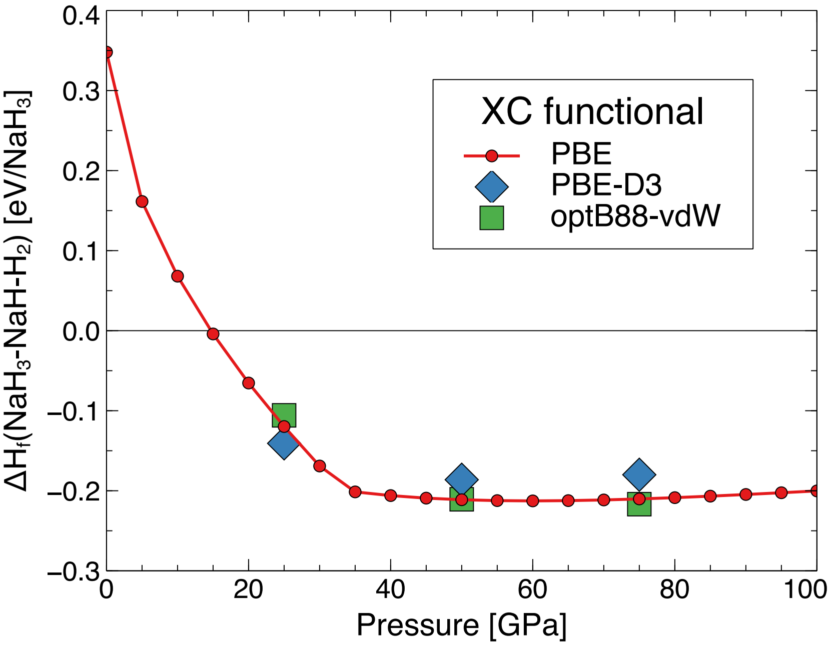

Figure S1. Formation enthalpy of NaH_3, computed from three different exchange-correlation (XC) functionals.

Figure S1 shows the enthalpy of formation of NaH_3 from $NaH+H_2$, comparing different exchange-correlation (XC) functionals. Both the semi-empirical D3 dispersion correction, and the fully nonlocal optB88-vdW functional show very little deviation from the PBE results. The latter are used throughout the main manuscript.

Figure S2 shows the Gibbs free energy of NaH_3 relative to $NaH+H_2$ for several relevant temperatures and pressures. For the Gibbs free energies $G(p,T) = E_{\mathrm{DFT}} + E_{\mathrm{ZPE}} + pV - TS$, the entropies S are approximated by harmonic vibrational entropies,

$$S \approx S_{\mathrm{vib}} = -k_B \int g(\omega) \log 1 - \exp -\frac{\hbar\omega}{k_B T} d\omega,$$

and zero-point vibrational energies

$$E_{\mathrm{ZPE}} = \frac{1}{2} \int \hbar\omega g(\omega) d\omega$$

are included. The phonon densities of state $g(\omega)$ are obtained from (3,3,3)/(4,4,4)/(4,2,3) supercells for the NaH-I/NaH-II/NaH_3-$Cmcm$ structures, respectively. For the H_2-$P6_3m$ structure, a non-diagonal supercell defined by the matrix ((3,-1,1),(0,-2,2),(0,-2,-2)) with 384 atoms was used.

The Gibbs free energies of formation for NaH_3 decrease with increased temperature, though the effects of ZPE and at room temperature are small. In the laser heating temperature range (1500-2100 K) the effects are more pronounced and (at this level of theory) formation of NaH_3 is favoured at higher, rather than lower, pressures.

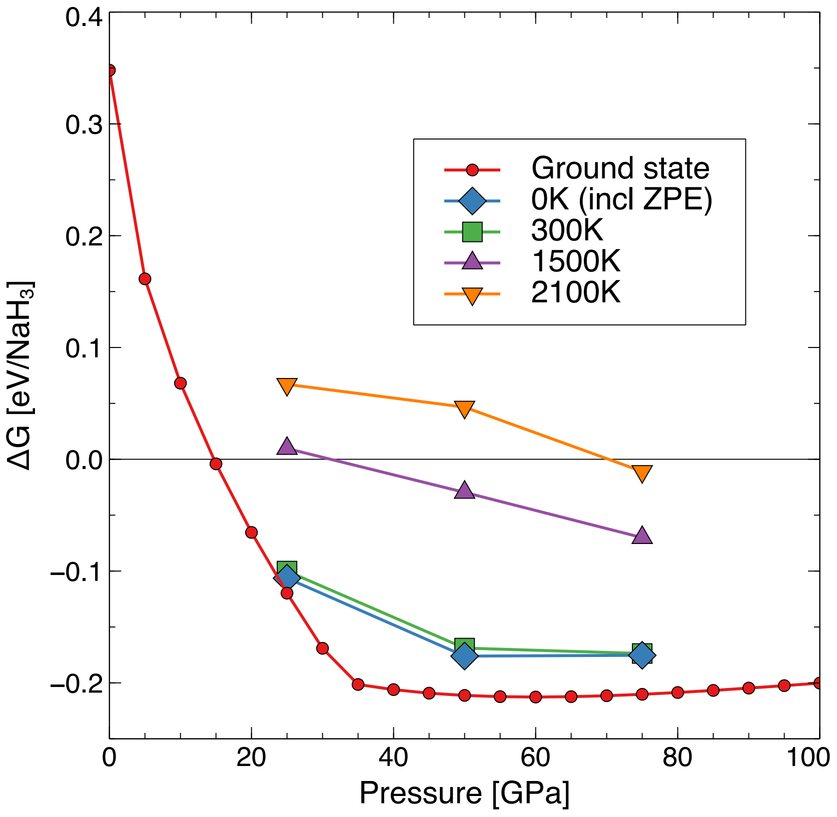

Figure S2. Relative Gibbs free energies of NaH_3 as function of pressure and temperature, from DFT-PBE results.

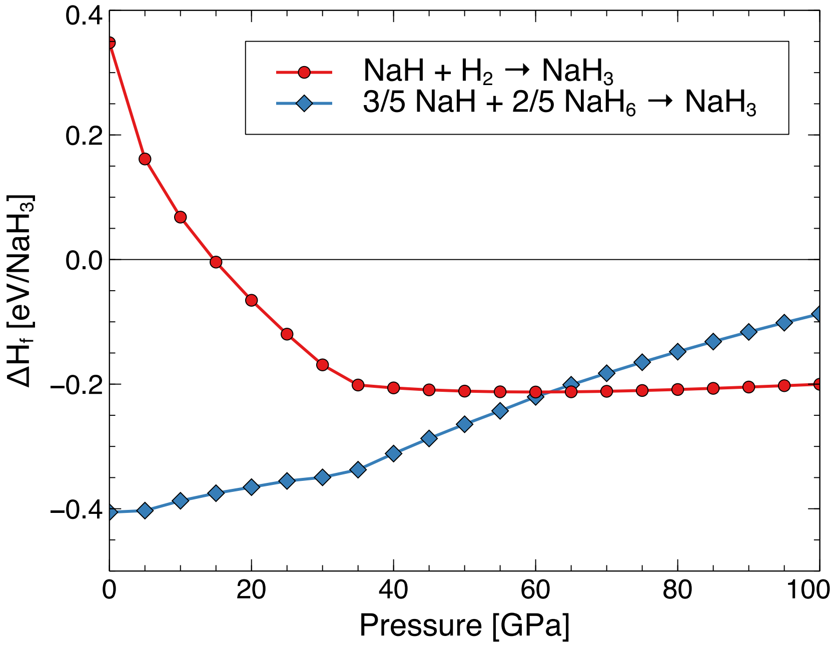

Figure S3. Reaction enthalpies of NaH_3 formation as labelled, as function of pressure, from ground state DFT-PBE results.

Figure S3 shows the formation enthalpy of NaH_3, firstly as reaction product of $NaH+H_2$, and secondly as reaction product of $\frac{3}{5}NaH+\frac{2}{5}NaH_6$, which considers the cubic NaH_6 phase recently studied by Chen *et al.* (New J. Phys. 23, 093007, 2021) and Shipley *et al.* (Phys. Rev. B 104, 054501, 2021). The former reaction becomes negative at 15 GPa, suggesting NaH_3 should form at or above that pressure. The latter is always negative; NaH_3 should always form NaH and NaH_6. The upwards kink in the formation enthalpies at 38 GPa is due to the NaH-I $\rightarrow$ NaH-II transition.

3 SUPPLEMENTARY EXPERIMENTAL DATA

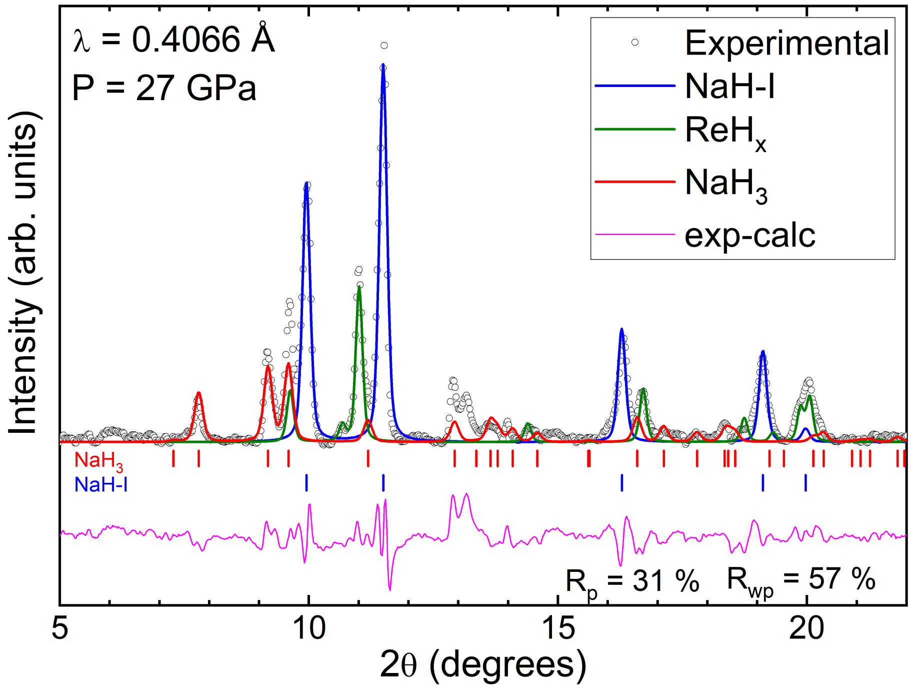

Figure S4. PXRD pattern of one of our samples after being laser heated at $\sim$ 30 GPa. Experimental data is shown as small circles. Red, blue and green solid lines represent the Rietveld refinement conrresponding to the NaH_3 ($Cmcm$, $a = 3.387$ Å, $b = 6.407$ Å, $c = 4.171$ Å), NaH-I (fcc, $a = 4.061$ Å) and ReH_x phases (hcp, $a = 2.798$ Å, $c = 4.371$ Å), as it is indicated in the legend. Preferred orientations in NaH-I were accounted using the March-Dollase model. The difference between the experimental and simulated pattern is depicted with a pink line. NaH_3 and NaH-II reflections are denoted with red and blue vertical ticks, respectively.

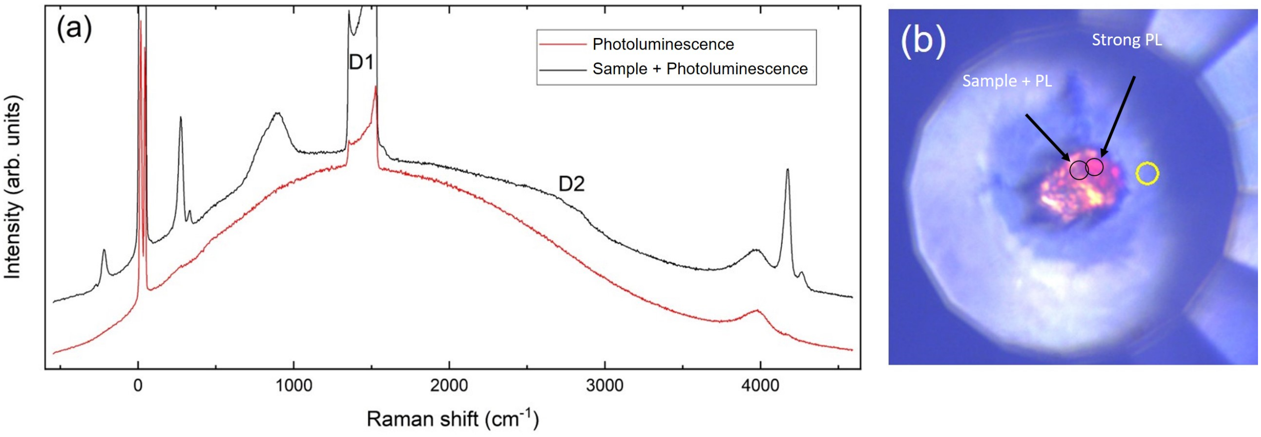

Figure S5. (a) Raman spectra of our sample at 75 GPa. Black and red lines represent measurements on and outside NaH_3, respectively. D1 and D2 correspond to the first and second order Raman modes of diamond. (b) Photo of the sample in transmitted and reflected light for XRD and Raman at 75 GPa (diamond culet size of 80 μm). Reddish areas, correspond to the regions where we observe the photoluminescence (PL). The positions at which the spectra shown in (a) were measured are indicated. The yellow circle it is an artefact, it does not mean anything here.

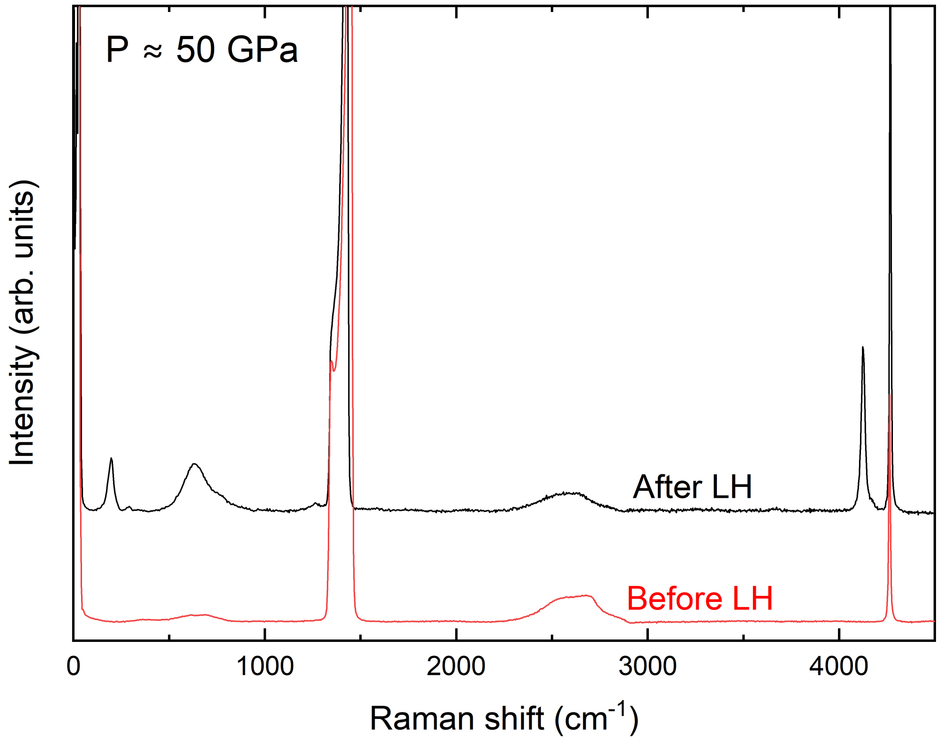

Figure S6. Raman spectra of our sample at ~ 50 GPa before and after laser heating (LH).
